# Dietary and nutritional strategies for reducing disease risk associated with antibiotic-induced dysbiosis

**DOI:** 10.1080/19490976.2026.2728330

**Published:** 2026-09-17

**Authors:** Hang Guo, Chengcheng Zhang, Ruimin Wang, Yijin Yang, Gang Wang, Shumao Cui, Wenwei Lu, Bo Yang, Xiaomei Lyu, Peijun Tian, Fengwei Tian, Jianxin Zhao, Qixiao Zhai

**Affiliations:** a State Key Laboratory of Food Science and Resources, Jiangnan University, Wuxi, Jiangsu, People's Republic of China; b School of Food Science and Technology, Jiangnan University, Wuxi, Jiangsu, People's Republic of China; c School of Food Science and Engineering, Hainan University, Haikou, People's Republic of China; d Shanghai Engineering Research Center of Food Microbiology, School of Health Science and Engineering, University of Shanghai for Science and Technology, Shanghai, People's Republic of China; e National Engineering Research Center for Functional Food, Jiangnan University, Wuxi, Jiangsu, People's Republic of China

**Keywords:** Antibiotic, dysbiosis, dietary pattern, nutritional intervention, disease risk, gut microbiota

## Abstract

Antibiotic exposure is a major driver of gut microbiota dysbiosis, and its effects extend beyond transient diversity loss. Persistent alterations in microbial composition and metabolites disrupt intestinal barrier integrity, immune homeostasis, and host metabolic signaling, thereby increasing the risk of chronic diseases. Here, we review the evidence linking antibiotic-induced dysbiosis to disease susceptibility and argue that post-antibiotic recovery should target microbial functions and metabolic network restoration. We also examine a range of nutritional strategies, including dietary adjustments, prebiotics, probiotics, and bioactive nutrients, for their capacity to support beneficial commensal bacteria, restore key microbial metabolites, and enhance the gut ecosystem resilience. However, inter-individual variability in response complicates the development of universal protocols, and limited long-term clinical outcome data make it difficult to determine the clinical value of these interventions. Integrating nutritional science, microbial ecology, and precision medicine will be essential for developing effective personalized interventions.

## 1. Introduction

The modern use of antibiotics emerged in the early 20th century with the discovery of penicillin by Alexander Fleming. This marked the beginning of effective systemic treatment for bacterial infections. The subsequent introduction of streptomycin and other antibiotics greatly reduced the burden of diseases such as pneumonia and tuberculosis, leading to substantial declines in mortality.^1^ Antibiotics not only transformed the management of infectious diseases but also made many advances in surgery, organ transplantation, and other areas of modern medicine possible.^2^ However, the achievements of antibiotics have also created their own crisis. Decades of overuse and inappropriate use have pushed antibiotic resistance to the forefront of clinical practice. It is a challenge that medicine must now confront directly. The development of resistance is mainly due to bacteria acquiring gene mutations or drug-resistant genes through horizontal gene transfer. The irrational use of antibiotics, such as unnecessary clinical prescriptions, insufficient dosage, premature discontinuation of drugs, and the use of antibiotics as agricultural growth promoters, has greatly accelerated this process.^3^ As a result, drug-resistant pathogens such as methicillin-resistant *Staphylococcus aureus* and multidrug-resistant *Mycobacterium tuberculosis* have become a global public health threat.^4^ Antibiotic resistance not only significantly increases the difficulty of treatment and medical costs, but also brings about a disturbing situation: common infections may once again put humanity in an era of no effective drugs.

The widespread use of antibiotics has accelerated antimicrobial resistance. At the same time, it inflicts often-ignored damage on the host's symbiotic microbiota. Most antibiotics have broad-spectrum antimicrobial properties, thus disrupting the structure and function of microbial communities in multiple ecological niches such as the gut and skin. This disruption manifests as decreased diversity, reduction of key symbiotic bacteria, and increase of opportunistic pathogens, leading to dysbiosis.^5^ This imbalance is closely related to host metabolic disorders and immune dysregulation. Epidemiological studies have shown that dysbiosis is associated with an increased risk of chronic diseases such as obesity, colitis, and allergic diseases.^6^ Antibiotic-induced host microbiome dysbiosis is particularly prominent in early life. These early disturbances can have long-term effects on host health through early life ecological imprinting.^7^ ^,^ ^8^ Furthermore, the natural recovery of certain microbial communities is often incomplete. Even after discontinuing the use of antibiotics, the structure and function of the gut microbiota may not return to the condition before treatment.^9^ This persistent dysbiosis may have a lasting effect on host physiology through cross-system pathways such as the gut-liver axis and metabolic-immune crosstalk.

To address the collateral damage to the gut microbiota caused by antibiotic treatment, two complementary intervention approaches have emerged. The upstream strategy focuses on preventing or mitigating dysbiosis at its source. Specific measures include prioritizing the use of narrow‑spectrum antimicrobials, avoiding drugs with anti‑anaerobic activity, and shortening the treatment course. For instance, fidaxomicin and similar antibiotics can treat *Clostridioides difficile* infection while sparing the commensal microbiota. They selectively target the pathogen and have a protective effect on the intestinal ecosystem.^10^ Novel phage-based precision antimicrobial agents further enhance targeting, as they eliminate specific pathogens without significantly disturbing the overall microbiome.^11^ The downstream strategy aims to promote restoration of microbial ecology and host homeostasis after microbiota damage occurs. As a non‑pharmacological practical approach, it helps mitigate the health risks associated with long-term antibiotic use. Upstream prevention strategies and downstream restorative measures complement each other, forming a relatively complete treatment paradigm, with this article focusing on the latter.

**Figure 1.**
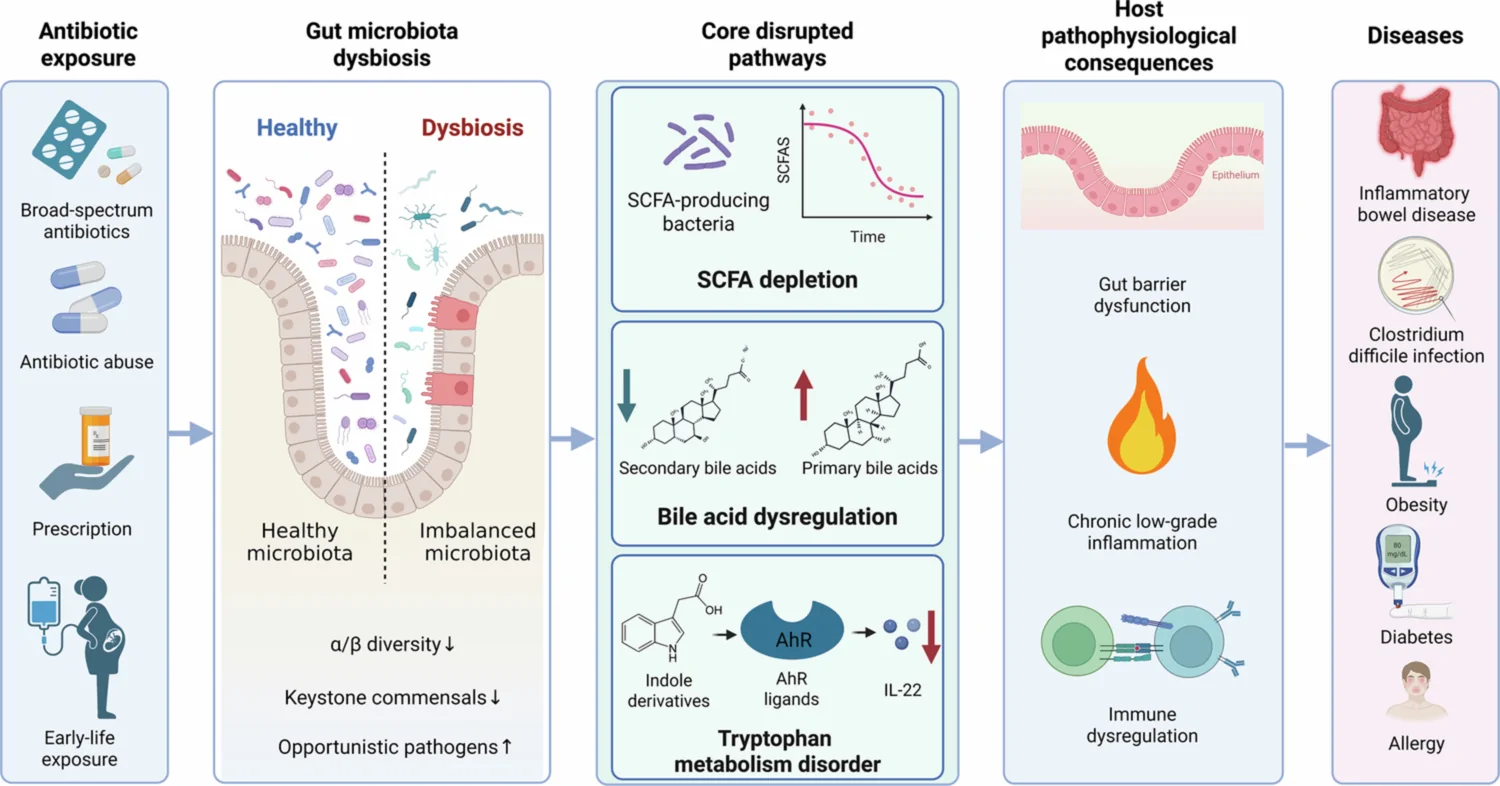
Antibiotic-induced gut microbiota dysbiosis, metabolic network disruption, and long-term disease risk.

This review explores two main aspects. The first concerns the mechanisms by which antibiotics disrupt gut microbiota homeostasis and increase the susceptibility to long-term diseases. We focus on studying the mechanistic evidence that can link gut microbiota dysbiosis with pathological outcomes such as inflammation, metabolism, and immunity. At the same time, we also explore the unique vulnerability observed during critical developmental stages, including early life. The second aspect addresses the pathways driven by this gut microbiota dysbiosis. Based on this, we critically evaluate emerging nutritional intervention strategies for mitigation. These approaches include both overall dietary patterns and more targeted interventions, such as probiotics, prebiotics, and specific bioactive nutrients. By integrating the evidence from animal and human cohort studies, we aim to transition the research results from the mechanistic level to the practical recommendation level, and provide clearer insights for clinicians and researchers to help them reduce the common disease burden after antibiotic treatment ( Figure 1).

## 2. Antibiotic‑induced gut dysbiosis and metabolic network disruption

### 2.1. Gut microbiota dysbiosis

Increasing evidence suggests that antibiotic exposure can significantly disrupt the gut microbiota, with effects that may persist even after treatment has ended. These effects typically manifest as reduced species diversity and altered community composition. In a widely cited longitudinal study, Dethlefsen and Relman^12^ examined the effects of ciprofloxacin on the gut microbiota of healthy adults. They showed that even short-term ciprofloxacin exposure caused rapid, profound, and highly individualized changes in the gut microbiota. In a substantial proportion of the subjects, the gut microbiota did not return to its pretreatment baseline after discontinuation of the drug. This disruption appeared to be more pronounced with broad-spectrum combination therapies. Palleja et al.^9^ reported data from a group of healthy volunteers who received a 4-day combination therapy of meropenem, gentamicin, and vancomycin. In that study, *α*-diversity required several months to return to baseline levels. Even after six months, *β*-diversity still differed significantly from baseline. This suggests that their structure and resistance may still be changing, and these changes will leave a lasting imprint. Direct comparisons of commonly used antibiotics also confirm this difference. Azithromycin has a longer half-life, which delays microbial recovery and leads to more pronounced compositional changes than levofloxacin.^13^

Antibiotic-induced gut microbiota dysbiosis is primarily driven by the differential susceptibility of gut bacterial groups to specific antibiotic classes. This differential susceptibility is determined by the presence or absence of antibiotic targets or inherent resistance genes. For example, *β*-lactam drugs primarily target the cell wall synthesis process of Gram-positive and Gram-negative bacteria in their active division phase, thereby eliminating susceptible bacteria while allowing inherently resistant taxa to survive.^14^ In contrast, fluoroquinolones act by inhibiting DNA gyrase and topoisomerase IV, thereby exhibiting antibacterial activity against both Gram-positive and Gram-negative bacteria. However, antibacterial efficacy varies among bacterial species owing to differences in target enzyme affinity, mutations in quinolone resistance-determining regions and the expression of multidrug efflux pumps.^15^ ^,^ ^16^ The depletion of susceptible commensal microbiota releases nutrients and opens up ecological niches, facilitating the rapid proliferation of previously disadvantaged bacterial populations. These bacteria are often intrinsically resistant or can readily acquire resistance through horizontal gene transfer.^17^ As a result, the combined genetic and ecological changes compromise colonization resistance and promote the rapid proliferation of opportunistic pathogens.

These mechanistic changes manifest as predictable alterations in microbiota composition. Evidence from systematic reviews and primary studies suggests that antibiotic exposure commonly reduces members of the Firmicutes and Actinobacteria phyla, particularly butyrate-producing and other short‑chain fatty acid (SCFA)-producing bacteria. Representative bacteria include *Faecalibacterium prausnitzii*, *Bifidobacterium*, *Roseburia*, and *Ruminococcus.* ^18–20^ Depletion of these beneficial bacterial communities is often accompanied by expansion of Proteobacteria, particularly the Enterobacteriaceae. Such shifts can weaken colonization resistance and thereby facilitate overgrowth or infection by opportunistic pathogens such as *C. difficile*. For example, oral vancomycin significantly reduces SCFA-producing bacteria such as *Coprococcus eutactus* and *Anaerostipes caccae*, while increasing the relative abundance of Enterobacteriaceae and *Enterococcus.* ^21^ In a pioneering study, Raymond et al. demonstrated that an individual’s baseline gut microbiota composition is a key determinant of antibiotic-induced microbiota remodeling and subsequent recovery dynamics. This finding indicates that even under the same treatment regimen, microbiota perturbation and recovery can differ substantially between individuals.^19^

Differences in routes of administration, intestinal exposure, and pharmacokinetic properties lead to different patterns of gut microbiota dysbiosis. Broad-spectrum antibiotics typically result in a more significant reduction in microbial diversity and obligate anaerobic commensal bacteria, while narrow-spectrum antibiotics usually produce only relatively limited ecological disturbances. Furthermore, antibiotics that enter the gut directly, especially those administered orally or poorly absorbed, may produce stronger luminal effects; systemically administered drugs can also alter the gut microbiota through bile secretion and intestinal excretion. To provide a systematic overview of class-dependent effects, Table 1 summarizes the mechanisms of action, representative drugs, target organs, effects on gut microbiota composition, and recovery dynamics for major antibiotic classes.

**Table 1.** Comparative effects of major antibiotic classes on gut microbiota and recovery dynamics.

Antibiotic Class	Representative Drugs	Mechanism of Action	Target organs/Gut exposure	Effects on Gut microbiota	Recovery Dynamics
β‑Lactams	Penicillins, cephalosporins, carbapenems	Inhibit bacterial cell wall synthesis	Systemic distribution with variable tissue penetration; gut exposure occurs through oral transit, incomplete absorption, and, for some agents, biliary excretion.	Reduction in diversity; depletion of Gram‑positive obligate anaerobes; reduced *Bifidobacterium* and *Lactobacillus*; transient expansion of Enterobacteriaceae. [22] Narrow-spectrum penicillins induce substantially less disturbance to the gut microbiota compared with cephalosporins or carbapenems. [23]	Near‑baseline recovery of most components within 30 days in healthy adults; [24] however, amoxicillin-associated changes may persist for up to 12 months in some individuals. [23]
Fluoroquinolones	Ciprofloxacin, levofloxacin, moxifloxacin	Inhibit DNA gyrase and topoisomerase IV	Wide tissue distribution; gut exposure via enterohepatic circulation and access to the intestinal lumen and mucosa.	Marked reduction in diversity; altered Bacteroidetes/Firmicutes ratio; enrichment of Enterobacteriaceae; Moxifloxacin depletes Ruminococcaceae more than other quinolones. [25]	Recovery begins within 1 week after cessation but is often incomplete; changes in community composition may persist for up to 1 year in some individuals. [12,23]
Macrolides	Azithromycin, clarithromycin	Inhibit protein synthesis (50S ribosomal subunit)	Biliary excretion, significant intestinal exposure; extensive tissue accumulation and long half‑life prolong gut exposure.	Reduced in diversity; significantly decreased *Bifidobacterium* and *Lactobacillus* species; increased Proteobacteria (e.g., *Escherichia coli*). [26]	Slower recovery than fluoroquinolones due to prolonged tissue half‑life; species richness reduced for up to 65 days after azithromycin; α‑diversity significantly lower for up to 4 years. [13,27]
Glycopeptides	Vancomycin	Inhibit cell wall synthesis (Gram‑positive specific)	Oral vancomycin is poorly absorbed, very high fecal concentration; acts primarily in the intestinal lumen.	Increased abundance of Proteobacteria and Verrucomicrobia; suppression of Firmicutes (mainly Clostridium) and elevation of Gram‑negative bacteria; lowest microbial diversity following vancomycin treatment. [28]	Up to 89% of abundant OTUs remain undetectable 22 weeks post‑cessation; richness does not return to baseline; [21] in mice, OTU composition does not return to baseline 1 month after withdrawal. [29]
Lincosamides	Clindamycin	Inhibit protein synthesis (50S ribosomal subunit)	Primarily systemic, but with pronounced downstream effects in the colon because of strong activity against anaerobic commensal communities.	Elimination of approximately one‑third of gut bacteria following short‑term oral clindamycin; reduced abundance of Actinobacteria and Firmicutes, with increased Bacteroidetes and Proteobacteria [24] ; more severe clindamycin‑induced dysbiosis compared to amoxicillin, characterized by 86–90% reduction in SCFA production. [30]	Gram‑positive anaerobes and *Bifidobacterium* do not normalize until month 12; OTU-level changes persist to month 4, with some effects still detectable at month 12. [31,32]
Nitroimidazoles	Metronidazole	Reduced under anaerobic conditions to reactive intermediates that damage DNA and cause strand breaks	Systemically distributed but preferentially active in anaerobic environments, leading to disproportionate effects on colonic anaerobe-rich communities.	Reduced in α‑diversity; decreased abundances of *Blautia*, *Fusobacterium*, *Turicibacter*, *Clostridium hiranonis*, and *Faecalibacterium*, with increased *Streptococcus* and *E. coli*; complete suppression of Ruminococcaceae; relatively mild impact on gut microbiota by nitroimidazoles compared to clindamycin and others. [25]	Rapid recovery and good resilience of microbiota following antibiotic withdrawal; canine model confirmed that while Shannon diversity remained reduced within 3 days after a 7‑day treatment, microbiota composition returned to pre‑treatment levels in all dogs within 6 months. [33]

### 2.2. Alterations in microbiota-derived metabolites

Previous reviews have extensively summarized the general role of microbial-derived metabolites in host immunity and metabolic homeostasis.^34^ ^,^ ^35^ In contrast, this section focuses specifically on how antibiotic exposure disrupts these metabolic pathways by depleting functionally important microbial groups, and the timing, selectivity, and potential long-term consequences of these changes. This is important because antibiotic-induced metabolic changes are distinctly different from those observed in other forms of dysbiosis. In many non-antibiotic environments, such as prolonged consumption of Western diets, obesity, or chronic inflammatory diseases, metabolic disorders typically develop gradually through persistent changes in nutrient supply, host inflammation levels, and mucosal physiology.^36^ In the context of antibiotic therapy, however, metabolic disorders often stem from a sudden reduction in specific functional groups, including fiber-fermenting anaerobic bacteria, bile acid-converting bacteria, and tryptophan-metabolizing commensal bacteria. Therefore, antibiotic-induced metabolic damage is typically characterized by rapid onset, severity correlated with antibiotic class, and delayed functional recovery. This damage may persist even after the microbial composition is restored, because the restoration of microbial diversity does not necessarily mean the restoration of key metabolites.

#### 2.2.1. Short‑chain fatty acid metabolism

Among microbiota-derived metabolites, SCFAs represent one of the most antibiotic-sensitive functional outputs of the gut microbiota. For example, oral vancomycin administration for 10 days in healthy adults significantly reduces fecal SCFA concentrations, including acetate, propionate, and butyrate.^37^ Choo et al. compared the effects of ciprofloxacin and vancomycin-imipenem combination therapy in mouse models. They found that both antibiotics significantly altered the fecal metabolome, but vancomycin-imipenem combination therapy caused a more pronounced reduction in propionate and other SCFAs, with levels remaining unrecovered even 9 days after treatment cessation. In contrast, ciprofloxacin-induced SCFA disturbances recovered relatively quickly after drug withdrawal.^38^ Recent study found that amoxicillin reduced total SCFAs in the mouse gut by approximately 76%, with partial recovery to a 73% reduction after two weeks of withdrawal. Azithromycin caused an approximate 70% reduction, which recovered to a 55% reduction after withdrawal. Clindamycin, on the other hand, caused the most severe and persistent SCFA depletion, with a decrease of 86%–90%, and no significant recovery was observed even two weeks after discontinuation. These differences were consistent with the degree of depletion and recovery capacity of SCFA-producing taxa, such as *Ligilactobacillus*, *Ruminococcaceae*, and *Lachnospiraceae.* ^31^ Such class-dependent differences indicate that antibiotic selection plays a decisive role in determining the severity of SCFA metabolic disruption. Importantly, this pattern differs from SCFA changes observed in other dysbiosis states. In diet-related dysbiosis, SCFA depletion typically occurs gradually due to chronic dietary fiber deficiency or altered substrate supply. Similarly, in obesity or inflammation-related dysbiosis, SCFA changes often occur concurrently with broader changes in host metabolism and immune status. In contrast, antibiotic-induced SCFA depletion more often reflects an acute disruption of microbial fermentation capacity, resulting from a reduction in key anaerobic producers and disruption of crossfeeding networks.

The pathological significance of this transient yet marked depletion lies not only in reduced SCFA concentrations, but in disruption of the metabolic networks responsible for their production and in loss of their physiological functions. In the colon of healthy individuals, butyrate is primarily produced via the butyryl-CoA:acetyl-CoA transferase pathway. A few microorganisms utilize the phosphotransbutyrylase-butyrate kinase pathway to complete this process.^39^ Antibiotic-induced loss of fermentative bacteria reduces carbohydrate fermentation levels, thereby decreasing the production of acidic metabolites, while simultaneously enhancing proteolytic fermentation and ammonia production. This metabolic shift increases the pH in the intestinal lumen, thereby altering the structure of the gut microbiota and indirectly inhibiting the propionate synthesis pathway dependent on succinate and propionyl-CoA.^40^

**Figure 2.**
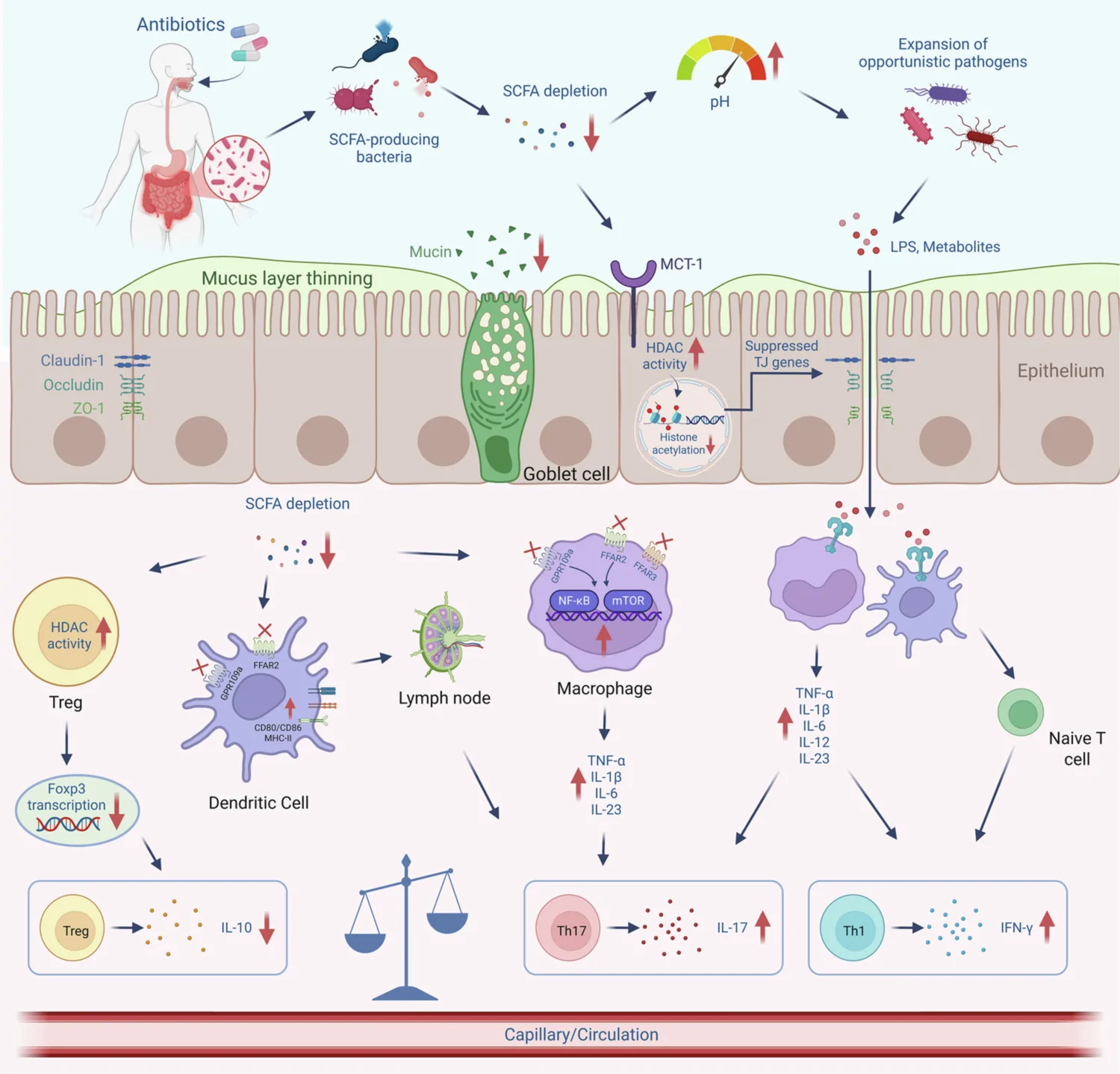
Antibiotic-induced SCFA depletion drives gut barrier dysfunction and immune dysregulation. This figure illustrates how antibiotics reduce SCFA levels, leading to intestinal barrier damage and immune imbalance. SCFA deficiency causes mucus thinning and tight junction loss, while also promoting Th17/Th1 responses over Tregs. These changes contribute to chronic inflammation and disease risk.

Functionally, butyrate is the main energy source for colon cells, so its deficiency impairs the metabolism of colonic epithelial cells. This impairment reduces the expression of tight junction proteins such as claudin-1, occludin, and ZO-1, ultimately damaging the integrity of the intestinal barrier.^41^ Butyrate and propionate can also exert immunomodulatory effects in two ways: first, by inhibiting histone deacetylases (HDACs),^42^ and second, by activating G protein-coupled receptors (such as GPR109A and FFAR2/3).^43^ Their downstream effects include inhibiting the mTOR signaling pathway, promoting regulatory T cell (Treg) differentiation, and reducing pro-inflammatory responses.^44^ Together, these interconnected pathways suggest that SCFA depletion is a key functional consequence of antibiotic-induced dysbiosis and an important mechanistic link between microbial disruption and the development of intestinal and systemic diseases (Figure 2).

#### 2.2.2. Bile acid metabolism

Antibiotic-related disruption of bile acid metabolism is mainly caused by the selective loss of bacterial taxa responsible for key biotransformation reactions. Antibiotic treatment can rapidly alter the balance among primary, conjugated, and secondary bile acids by eliminating bacterial taxa responsible for key biotransformation steps.^45^ Since these functions are primarily concentrated in specific anaerobic gut commensals, the extent of bile acid dysregulation may vary depending on the antibiotic spectrum. A recent study by Yang et al. showed following 3-day administration of four different antibiotics, moxifloxacin and metronidazole significantly inhibited the production of secondary bile acids, while cefixime and clarithromycin had only minor effects. Mechanistically, this difference is attributed to the varying capacities of these antibiotics to deplete Ruminococcaceae (the primary bacterial family harboring 7α-dehydroxylase).^25^ In human studies, broad-spectrum vancomycin increased the fecal conjugated/secondary bile acid ratio by nearly 100‑fold from baseline in patients with *C. difficile* infection (CDI), whereas narrow-spectrum ridinilazole maintained this ratio close to baseline levels. This difference was attributed to the depletion of secondary bile‑acid‑producing *Bacteroidales* and *Clostridiales* bacteria by vancomycin, whereas these microbial populations remained largely stable in the ridinilazole group.^46^ In summary, these observations suggest that bile acid dysregulation following antibiotic treatment is not uniform, but rather depends on the pharmacological spectrum of the antibiotic and the microbial function it disrupts.

The mechanistic basis for these changes lies in the antibiotic-induced depletion of bacteria that mediate the conversion of secondary bile acids. Secondary bile acids, such as deoxycholic acid (DCA) and lithocholic acid (LCA), are produced via 7α-dehydroxylation.^47^ This process is carried out by an enzyme system encoded by the bile acid-inducible (bai) operon. The bai operon is conserved in several *Clostridium* species, such as *Clostridium scindens* and *Clostridium hylemonae*. Broad-spectrum antibiotics with anti-anaerobic activity (such as vancomycin, clindamycin, and cefoperazone) can rapidly deplete these functional microbial populations, leading to a loss of secondary bile acids and accumulation of primary bile acids.^48^ Furthermore, primary bile acids such as taurocholate are potent inducers of *C. difficile* spore germination, whereas secondary bile acids typically inhibit the growth of *C. difficile* vegetative cells. Consequently, the depletion of secondary bile acids creates a favorable environment for spore germination and pathogen proliferation, providing a mechanistic explanation for the strong association between antibiotic exposure and CDI.^49^

In addition to their metabolic functions, certain microbial-derived secondary bile acids also function as important immunomodulatory molecules. For example, 3-oxolithocholic acid (3-oxoLCA) and isolithocholic acid (isoLCA) can directly bind to and inhibit retinoic acid-related orphan receptor γt (RORγt), thereby inhibiting the differentiation of T helper cells 17 (Th17).^50^ Another study showed that the secondary bile acid isoallolithocholic acid (isoalloLCA) can promote the differentiation of Treg cells by increasing mitochondrial reactive oxygen species (ROS) levels, a process dependent on nuclear receptor subfamily 4 A member 1 (NR4A1).^51^ Mouse and human studies have shown that glycodeoxycholic acid (GDCA) can promote the production of interleukin-22 (IL-22) by intestinal type 3 innate lymphoid cells (ILC3), which helps maintain epithelial barrier homeostasis. This pathway is associated with metabolic and inflammatory diseases and is linked to the Takeda G protein-coupled receptor 5 (TGR5) signaling pathway.^52^ Overall, antibiotic-induced bile acid remodeling can disrupt host metabolic and immune homeostasis.

#### 2.2.3. Tryptophan metabolism

Tryptophan metabolism is highly susceptible to disruption by antibiotics, as the conversion of dietary tryptophan into biologically active microbial metabolites relies on a limited set of commensal bacterial species.^53^ Metabolomics studies have shown that ciprofloxacin treatment significantly reduces serum levels of indole-3-propionic acid (IPA), a key tryptophan-derived aryl hydrocarbon receptor (AhR) ligand produced by gut bacteria.^54^ Perdijk et al. further demonstrated that early‑life antibiotic exposure reduces IPA to undetectable levels, persistently impairs airway epithelial mitochondrial function, and exacerbates allergic airway inflammation in adulthood. These effects persist even after the microbiota recovery and can be reversed by IPA supplementation, suggesting that specific antibiotics exert lasting impacts on host metabolism and immune function by depleting IPA‑producing microbiota.^7^ A broad-spectrum antibiotic cocktail (containing vancomycin, neomycin, metronidazole, and ampicillin) can significantly inhibit the production of IPA by gut microbiota, whereas fecal microbiota transplantation restores its levels.^55^ Since microbial tryptophan catabolism is concentrated in specific anaerobic taxa, antibiotics with strong anti‑anaerobic activity disrupt this pathway more profoundly than narrow‑spectrum antibiotics.

Tryptophan is metabolized primarily through three pathways: direct conversion by microorganisms into ligands of AhR, such as IPA, indole-3-carboxaldehyde (IAld), and 3-indoleacrylic acid (IA); the host kynurenine pathway and the serotonin pathway.^56^ Certain bacteria, particularly *Clostridium sporogenes* and *Lactobacillus* spp., can produce AhR agonizts such as IPA, indole-3-acetic acid (IAA), and IAld. These agonizts play a crucial role in maintaining intestinal barrier integrity and immune homeostasis.^57^ Therefore, the loss of these metabolites after antibiotic treatment has direct functional consequences: weakened AhR signaling can impair epithelial barrier function, reduce IL-22-mediated mucosal defense, and promote a pro-inflammatory immune environment.^58^ It is noteworthy that the acute depletion of AhR ligands following antibiotic exposure differs from the gradual decline observed in chronic inflammatory diseases. In inflammatory bowel disease (IBD), the reduction in AhR signaling is a process that lasts months, driven by chronic inflammation and altered gut microbiota composition, with serum IPA levels fluctuating with disease activity.^59^ In contrast, antibiotics cause abrupt loss of AhR ligands by directly depleting specific microbiota, typically occurring within days of treatment initiation.

Within the AhR pathway, cytochrome P450 family 1 subfamily A member 1 (CYP1A1) participates in a negative feedback loop that helps control the availability of AhR ligands. Excessive CYP1A1 activity can accelerate the degradation of AhR ligands, thereby limiting sustained AhR signaling, which has potential implications for mucosal immune homeostasis. Increasing the supply of ligands from dietary or microbial sources could partially counteract this effect.^60^ In animal models, tryptophan or specific probiotics such as *Bifidobacterium bifidum* have alleviated dextran sulfate sodium (DSS)-induced colitis, at least in part by restoring AhR ligand production.^61^ Beyond CYP1A1-mediated feedback, microbiota-derived tryptophan metabolites can influence AhR signaling by modulating cytochrome P450 activity and the phosphorylation of signal transducer and activator of transcription 3 (STAT3).^60^ For example, IPA inhibits indole-3-sulfate (ISA)-induced CYP1A1 expression and thus prevents AhR overactivation. Dysregulation of the tryptophan-AhR axis has been increasingly linked to gut microbiota disturbances in neuropsychiatric and metabolic disorders.^62^ In summary, antibiotic-induced tryptophan metabolism disorder not only depletes AhR ligands required to maintain the intestinal barrier integrity and immune homeostasis, but also induces an acute immune vulnerable state that may increase susceptibility to disease.

## 3. Epidemiological and mechanistic links between antibiotic exposure and long‑term disease risks

### 3.1. *Clostridioides difficile* infection and inflammatory bowel disease

Antibiotic exposure is strongly associated with an increased risk of gastrointestinal disease. This association is thought to be mediated largely by antibiotic-induced disruption of gut microbiota structure and function. Such disruption weakens colonic colonization resistance and thereby permits the expansion of opportunistic pathogens. One of the clearest clinical consequences of this process is CDI. Most patients with healthcare-associated or community-acquired CDI have a history of antibiotic exposure before the onset of infection.^63^ In a large-scale clinical study, Tartof et al. showed that in-hospital use of third-generation cephalosporins, fluoroquinolones, and lincosamides substantially raised the risk of CDI. If patients were exposed to three or more classes of antibiotics, the risk increased by more than tenfold.^64^ Buffie et al. addressed this in mice. A single dose of clindamycin was sufficient to cause a reduction in gut microbial diversity for at least 28 days. Approximately 90% of the native bacteria disappeared, while opportunists such as Enterobacteriaceae took their place. In the wake of this collapse, the mice remained highly susceptible to *C. difficile* infection, and this susceptible state persisted for at least 10 days following clindamycin treatment.^65^ Using a multi-omics approach, Theriot et al. showed that antibiotic treatment sharply raised levels of primary bile acids such as taurocholic acid in the gut, which are potent germination signals for *C. difficile* spores.^66^

Evidence from population studies supports a link between antibiotic exposure and increased IBD risk. In a Swedish nationwide case-control study, the use of antibiotics was associated with an increased risk of developing IBD (aOR = 1.88), and this association persists even when comparing siblings. This indicates that shared genetic background alone cannot explain this finding.^67^ Similar results were also obtained in a Danish cohort, where this association was more pronounced in people over 40 years old and those who had received antibiotic treatment for gastrointestinal pathogens.^67^ A 2024 meta-analysis pooled data from 28 studies. The pooled results suggested that antibiotic use was linked to an approximately 40% increase in IBD risk (odds ratio [OR] = 1.41), with evidence of a dose-response relationship. The strength of the association differed across antibiotic classes. Metronidazole and quinolones stood out, with significantly higher associated risks (ORs of 1.70 and 1.56).^68^ These findings are generally thought to reflect antibiotic-induced disruption of the gut microbiota. Such changes promote the expansion of potentially pathogenic taxa, impair the intestinal mucus barrier, and favor the development of a pro-inflammatory microenvironment.^69^ Emerging evidence also suggests that antibiotic-induced microbial dysbiosis may have intergenerational effects. When dysbiosis occurs in the parental generation, it may be vertically transmitted to offspring. The result in offspring is amplified inflammation and more severe colitis, perpetuating a cycle of disease susceptibility across generations.^70^

### 3.2. Obesity and diabetes

A consistent body of epidemiological evidence now links early-life antibiotic exposure to an elevated risk of obesity in later childhood and adulthood. In a systematic review by Miller et al., children treated with antibiotics during infancy showed a pooled OR of 1.05 for overweight or obesity. The association was stronger in male infants and in those exposed to broad-spectrum antibiotics, including macrolides.^71^ A meta-analysis of 23 observational studies involving more than 1.25 million children found that early childhood antibiotic use was associated with a 14% higher risk of overweight/obesity.^72^ Large birth cohort studies have also linked antibiotic exposure before age 2 to childhood obesity. The association appears to be dose dependent, with risk increasing as the frequency of exposure and breadth of antibiotic coverage increase.^73^ Antibiotics likely promote obesity by disrupting gut microbiota structure and function, leading to dysbiosis and altered host energy metabolism. Schell and Carmody proposed an “energy framework” in which the metabolic effects of antibiotic-induced microbiome perturbations depend on dose. High-dose exposure sharply reduces microbial density, creating a transient negative energy balance. Paradoxically, however, this exposure simultaneously increases the long-term risk of obesity. This effect is thought to involve reduced SCFA production and altered energy harvest efficiency.^74^ Early-life antibiotic exposure also shifts the gut Firmicutes-to-Bacteroidetes ratio. This shift may increase energy harvest and promote adipose tissue accumulation.^75^ Antibiotics also reduce the abundance of beneficial SCFA-producing bacteria, especially butyrate-producing taxa. This change can weaken the intestinal barrier and increase gut permeability. Once the barrier is damaged, lipopolysaccharides (LPS) from gut bacteria can move into the bloodstream. When endotoxins leak into the bloodstream, they trigger a persistent low-level inflammatory response and exacerbate metabolic problems such as insulin resistance and ectopic fat accumulation.^76^

The gut microbiota is established during the early stages of life, and this period is precisely when exposure to antibiotics may have long-term effects. The dysbiosis that follows can derail normal immune development and metabolic regulation, thereby increasing the risk of developing type 1 diabetes (T1DM) and type 2 diabetes (T2DM). In a Swedish national study, children who received antibiotics during infancy were 19% more likely to develop T1DM by age 10.^77^ For T2DM, a systematic review by Zhou et al. reported a pooled OR of 1.16 among individuals with prior antibiotic exposure. Among individuals aged 50 years or older, the estimate was slightly higher (OR = 1.17).^78^ Risk also appears to vary by antibiotic class. Broad-spectrum antibiotics have been more strongly linked to T1DM,^79^ whereas beta-lactams and macrolides show stronger associations with T2DM.^78^ Mechanistic studies suggest that antibiotic-induced perturbation of the gut microbiota can alter the development of both immune and metabolic pathways.^80^ In T1DM, antibiotic-induced dysbiosis can serve as a decisive environmental trigger. Animal models show that this dysbiosis impairs at least three protective pathways: it depletes SCFA-producing bacteria, weakens the microbiota-mediated induction of regulatory T cells (Tregs), and disturbs the Th17/Treg balance within the intestinal lamina propria. Together, these changes impair immune tolerance and promote autoimmune injury to pancreatic islets.^81^ Some antibiotics, such as vancomycin, may accelerate T1DM progression by promoting the differentiation of pro-inflammatory T cells. These cells then produce interleukin-17 (IL-17) and interferon-*γ* (IFN-*γ*) in the spleen and pancreatic lymph nodes. The process is driven by the myeloid differentiation primary response 88 (MYD88)-dependent signaling pathway.^82^ In T2DM, antibiotic-related shifts in the microbial community may disturb SCFA metabolism and impair intestinal barrier integrity. Such changes may increase circulating LPS and sustain low-grade inflammation, consistent with metabolic endotoxemia. These processes are thought to contribute to the development of insulin resistance.^83^

### 3.3. Allergic diseases and immune dysregulation

Epidemiological studies consistently link antibiotic exposure during early life, including the prenatal period and infancy, to a higher risk of allergic disease. These conditions are largely associated with T helper 2 (Th2)-skewed immune responses and mainly include asthma or wheeze, eczema or atopic dermatitis (AD), and food allergy. The causal mechanisms remain incompletely understood, but the associations rest on a large body of prospective cohort studies and meta-analyzes. Zhong et al. conducted a meta-analysis comprising 26 observational studies drawn from populations across Europe, North America, and Asia. Prenatal antibiotic exposure was associated with a higher risk of childhood asthma or wheezing (OR = 1.29), and this association was stronger after age three. It was also associated with a higher risk of eczema or AD (OR = 1.62).^84^ No statistically significant association emerged for food allergies, possibly because of the small number of available studies (only three). The authors also noted that antibiotic exposure during infancy may confound, and possibly exaggerate, the association attributed to prenatal exposure. A systematic review by Cait et al. covering 27 studies provided additional support. It showed that prenatal antibiotic exposure was associated with a higher risk of childhood asthma (relative risk [RR] = 1.28), with evidence of a dose–response relationship as risk increased with multiple antibiotic courses.^85^ The same review reported a similar risk estimate for AD (RR = 1.28). Similarly, data from the Danish National Birth Cohort linked prenatal antibiotic exposure to a higher risk of AD at 18 months of age (OR = 1.45).^86^ In the rural PASTURE cohort, prenatal antibiotic exposure was associated with infantile AD and food allergies, but not with asthma or allergic rhinitis.^87^ Wypych et al., drawing on both retrospective and prospective studies, concluded that antibiotic exposure during infancy is associated with a higher risk of asthma. Depending on the timing of exposure, the estimated ORs ranged from 1.19 to 3.97. Children exposed before six months of age faced the greatest risk.^88^

The hygiene hypothesis offers one possible explanation: limited microbial exposure early in life may steer immune development toward a Th2-dominant pattern and increase susceptibility to immunoglobulin E (IgE)-mediated allergic responses.^89^ ^,^ ^90^ Maternal antibiotic exposure during pregnancy, for instance to vancomycin, has been shown in animals to disrupt multiple aspects of the offspring’s immune development. In animal models, maternal antibiotics reduce the colonization of the fetal gut by beneficial microbes, lower SCFA output, impair Treg induction, and push the immune system toward Th2 and Th17 responses.^91^ Alhasan et al. extended these findings in a mouse model of prenatal exposure: the offspring born to mother mice that received antibiotic treatment suffered from more severe asthma symptoms, and the effect was dose-dependent. The fundamental problem seems to lie in the interruption of the vertical microbial transmission between the mother and the offspring, which compromised pulmonary immune tolerance in the offspring.^92^ Human studies point in a similar direction. When mothers receive antibiotics during pregnancy, the infant gut microbiota often shifts, with protective genera such as *Bifidobacterium* and *Lactobacillus* becoming less abundant. This early dysbiosis has been linked to immune dysregulation and a greater risk of allergic disease.^93^ Tapiainen et al. showed that antibiotic exposure during labor and the postpartum period disrupts the normal maturation of the infant gut microbiota. This disruption promotes low-level inflammation, which is the basis of allergic reactions.^94^ Prescott et al. emphasized that perinatal antibiotic exposure rewires early microbial colonization and can derail normal immune development, raising the risk of atopic outcomes^93^(Table 2).

**Table 2.** Clinical studies assessing the impact of antibiotic exposure on disease risk.

Study	Disease type	Date (location)	Study design	Sample size	Antibiotic exposure timing	Antibiotic data source	Key findings
Faye et al. [67]	IBD (UC & CD)	2020 (Denmark)	Population-based cohort	6,104,245	Age ≥ 10 years (2000–2018)	Danish National Prescription Register	Any antibiotic exposure increased IBD risk across all ages (IRR 1.28–1.47). Dose-response: ≥ 5 courses IRR 1.69–2.12. Highest risk 1–2 years post-exposure, especially with GI-targeting antibiotics.
Nguyen et al. [95]	IBD (UC & CD)	2020 (Sweden)	Nationwide case-control	141,809 (23,982 cases)	Cumulative use up to 1 year before diagnosis	Swedish Prescribed Drug Register	Any antibiotic uses increased IBD risk (aOR 1.88). Dose-response: ≥ 3 courses aOR 1.55. Broad-spectrum antibiotics showed stronger association. Association persisted in sibling comparison (aOR 1.35).
Oh et al. [96]	IBD (UC & CD)	2023 (South Korea)	Population-based case-control	411,798 (68,633 cases)	2–5 years before diagnosis; first year of life (pediatric)	Korean NHIS database	Any antibiotic exposure increased IBD risk (AOR 1.24). Dose-response ≥ 20 prescriptions AOR 2.00. Broad-spectrum increased risk; narrow-spectrum did not. Early-life exposure associated with childhood IBD (OR 1.51).
Boven et al. [97]	CDI	2026 (Sweden)	Population-based case-control	398,080 (42,921 cases)	30 days before index date	Swedish Prescribed Drug Register	Highest CDI risk: lincosamides (aOR 31.4), combination penicillins (aOR 19.8), sulfonamides/trimethoprim (aOR 12.1), cephalosporins (aOR 13.8).
Webb et al. [98]	CDI	2020 (USA)	Retrospective cohort	506,068 admissions (2,356 cases)	60 days prior to index admission	Intermountain Healthcare EHRs	Prior antibiotic use dominant risk factor: each day increased odds by 12.8%. Highest risk: cephalosporins, carbapenems, fluoroquinolones.
Dingle et al. [99]	CDI	2017 (England)	Observational	4,045 isolates	1998–2014 (national); 2006–2013 (Oxfordshire)	IMS Health; whole genome sequencing	CDI decline (~80%) driven by elimination of fluoroquinolone-resistant isolates (from 67% to 3%; aIRR 0.52). Susceptible isolates unchanged. Fluoroquinolone restriction appears causal.
Aversa et al. [100]	Obesity	2021 (USA)	Population-based cohort	14,572 children (10,220 exposed)	First 2 years of life	Rochester Epidemiology Project	Early antibiotic exposure increased childhood obesity risk (HR 1.20). Dose-response: ≥ 5 prescriptions HR 1.37–1.45. Cephalosporins, macrolides, penicillins each associated.
Scott et al. [73]	Childhood obesity	2016 (UK)	Retrospective cohort	21,714 children	Before age 2 years	THIN electronic medical records	Antibiotic exposure before age 2 increased obesity at age 4 (aOR 1.21). Dose-response: 3–5 courses aOR 1.41, ≥ 6 courses aOR 1.47. Anti-anaerobic antibiotics showed dose-dependent association.
Clausen et al. [79]	Type 1 diabetes	2016 (Denmark)	Nationwide cohort	858,201 singletons (1,503 cases)	First 2 years of life	Danish Register of Medicinal Product Statistics	Broad-spectrum antibiotics in first 2 years increased T1D risk (HR 1.13), modified by delivery mode: C-section HR 1.70–1.63, vaginal HR 1.05. Narrow-spectrum not associated.
Mikkelsen et al. [101]	Type 2 diabetes	2015 (Denmark)	Population-based case-control	1,534,511 (170,504 cases)	1995–2012 (excluding 6 months before index)	Danish National Prescription Registry	≥ 5 antibiotic prescriptions increased T2D risk (aOR 1.53). Dose-response observed. Narrow-spectrum (aOR 1.55) and bactericidal (aOR 1.48) slightly higher risk than broad-spectrum (aOR 1.31).
Patrick et al. [102]	Childhood asthma	2020 (Canada)	Population-based + prospective (CHILD)	4–7 million (population); 2,644 children (CHILD)	First year of life	BC PharmaNet; CHILD questionnaires	Decrease in antibiotic prescribing correlated with decreased asthma incidence (Spearman's r = 0.81). Infant antibiotic use strongly associated with asthma (aOR 2.15).
Miter et al. [103]	Allergic diseases	2018 (USA)	Retrospective cohort	792,130 children	First 6 months of life	Military Health System database	Antibiotics in first 6 months increased risk of asthma (aHR 2.09), allergic rhinitis (aHR 1.75), anaphylaxis (aHR 1.51), allergic conjunctivitis (aHR 1.42), food allergy (aHR 1.14), atopic dermatitis (aHR 1.18), and others.
Timm et al. [86]	Atopic dermatitis	2017 (Denmark)	Population-based cohort	62,560 mother-child pairs	Prenatal (1st-2nd, 3rd trimester, or both)	Telephone interviews (DNBC)	Prenatal antibiotics throughout pregnancy increased AD at 18 months in children of atopic mothers (aOR 1.45).

## 4. Dietary and nutritional intervention strategies for alleviating antibiotic-induced dysbiosis and reducing disease risks

Antibiotic-induced gut microbiota dysbiosis can lead to a variety of pathophysiological consequences. These consequences strongly demonstrate the importance of nutritional strategies in practice. The principle is based on a simple truth: dietary interventions can alter the nutrients available to the microbes, change the gut's metabolic environment, and modulate the host's inflammatory response. The following chapters will explore specific nutritional strategies, from broad dietary patterns to more targeted approaches, such as interventions oriented towards short-chain fatty acids (SCFAs). We will discuss how to utilize these strategies to combat antibiotic-induced gut microbiota dysbiosis and reduce its long-term health consequences.

### 4.1. Dietary patterns

#### 4.1.1. High‑fiber diet

A high-fiber diet is essentially a substrate-based intervention, the characteristics of which are determined by the quantity, type, and fermentation properties of microbiota-accessible carbohydrates. Fermentable fibers and resistant starches are the principal substrates for anaerobic fermentation by colonic microbes. The SCFAs released by this process are central regulators of intestinal homeostasis.^104^ Acetate and propionate take part in lipid and glucose metabolism, while butyrate fuels colonocytes and helps maintain tight junction proteins. All three SCFAs also support the differentiation of Tregs.^105^ Antibiotics can cause a sharp decline in the number of these SCFA-producing gut bacteria. Therefore, a high-fiber diet can promote the growth of a few specific bacterial groups, thereby restoring the fermentation process that produces SCFAs in the whole microbiome.

In addition to directly influencing host physiology, SCFAs play a key role in shaping the intestinal luminal environment. When antibiotic use leads to a reduction in SCFA-producing bacteria, colonic epithelial cell metabolism is impaired and luminal oxygen levels rise. This creates a favorable environment for the overgrowth of Proteobacteria. A multi-omics study revealed how dietary fiber protects the gut microbiota during antibiotic-induced dysbiosis.^106^ Fiber suppressed the expansion of Proteobacteria and helped restore Firmicutes and Bacteroidetes by lowering the intestinal redox potential and promoting a more anaerobic environment. This effect stems from the oxygen consumption during the fermentation of fiber into SCFAs. The oxygen-consuming effect is particularly important after antibiotic treatment, as a typical feature of post-antibiotic gut microbiota dysbiosis is an increase in the ratio of aerobic to facultative anaerobes. Another study showed that pectin supplementation promoted the enrichment of anaerobic bacteria from the family Lachnospiraceae, enhanced microbial reductive metabolism, and inhibited the oxidative pathway of catabolism. Importantly, transplantation of fecal microbiota from pectin-treated donors reproduced the accelerated reduction in redox potential and Enterobacteriaceae load, indicating that the effect of pectin on intestinal redox potential is mainly achieved by remodeling the gut microbiota rather than its inherent antioxidant properties.^107^

**Figure 3.**
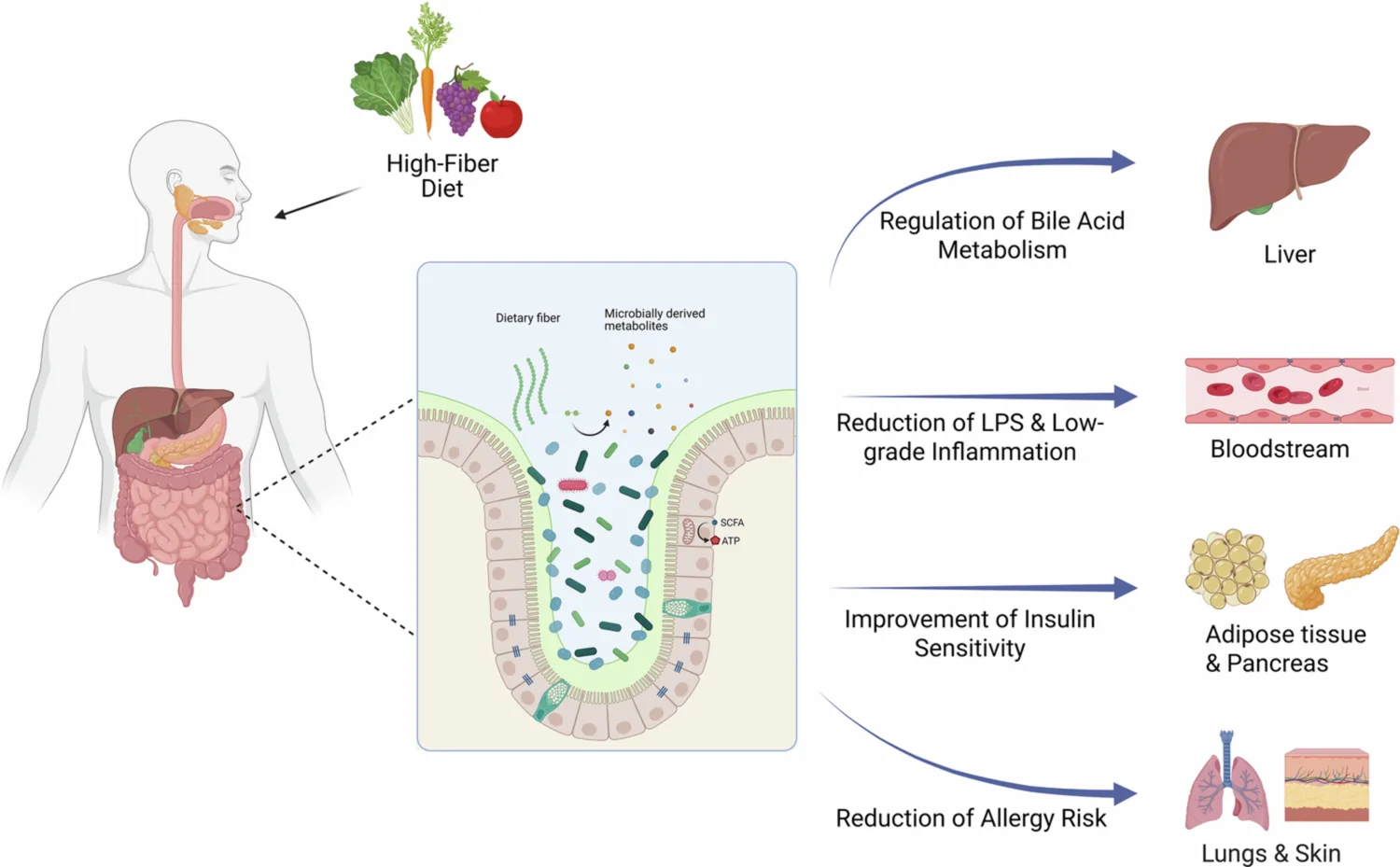
Systemic benefits of high-fiber diet in mitigating post-antibiotic disease risk.

From the perspective of diet, a diet rich in whole grains, legumes, vegetables, fruits, nuts, seeds, inulin-containing foods, and resistant starch foods are beneficial for promoting gut recovery after antibiotic treatment.^108^ The effectiveness depends on the type and fermentability of dietary fiber, as well as the individual's baseline gut microbiota. For individuals with sensitive guts or functional symptoms, consuming a large amount of fermentable dietary fiber suddenly may cause bloating or flatulence, which often leads to poor adherence.^109^ A simple and practical strategy is to gradually increase dietary fiber intake and adjust it according to each individual's actual tolerance (Figure 3).

#### 4.1.2. Mediterranean diet

The Mediterranean diet is a holistic dietary pattern rather than an intervention based solely on fiber intake. Beyond fermentable carbohydrates, it is characterized by a high intake of polyphenol-rich plant-based foods, monounsaturated fatty acids (MUFAs), and omega-3 polyunsaturated fatty acids (*ω*-3 PUFAs).^110^ These components shape both the gut microbial ecosystem and the host's physiological state. Healthy fats from olive oil, nuts, and fish help optimize the host's lipid profile. Their rich phytochemicals also relieve oxidative stress and chronic inflammation.^111^ ^,^ ^112^ In the context of antibiotic exposure, compared to simply increasing fiber intake, this dietary pattern may provide a more favorable ecological and host metabolic environment for the recovery of the microbiota.

Consistent evidence from large-scale cohort studies suggests that higher adherence to the Mediterranean diet is associated with greater gut microbial alpha diversity and enrichment of species such as *Barnesiella*, *Butyricicoccus*, *Agathobacter*, and *Bacteroides*, which are typically associated with SCFA production and gut homeostasis.^113^ Systematic reviews and meta-analyzes further suggest that the Mediterranean diet improves biomarkers of oxidative stress and early inflammation, supporting the view that its benefits extend beyond single nutrients to encompass broader host-microbiome interactions.^110^ In the context of antibiotic-induced gut microbiota dysbiosis, the main components of the Mediterranean diet may promote gut microbiota recovery through complementary mechanisms. The fermentation of dietary fiber provides the foundation for the production of SCFAs, while polyphenols and *ω*-3 polyunsaturated fatty acids may provide additional support on this basis.^114^ Dietary polyphenols can inhibit oxidative stress and modulate microbial competition. Furthermore, following metabolism by the gut microbiota, certain polyphenols generate bioactive small molecules that exert anti-inflammatory and barrier-protective effects.^115^ By reducing the production of inflammatory mediators and maintaining the stability of cell membranes, it can help counteract the pro-inflammatory environment caused by antibiotics.^116^

As a suitable long-term dietary approach, the Mediterranean diet is appropriate for the recovery period after antibiotic treatment. It helps to restore the ecological functions related to *Bifidobacterium* and certain butyrate-producing bacteria.^117^ At the same time, by improving bile acid metabolism and the overall metabolic environment of the host, the Mediterranean diet may reduce the risk of subsequent obesity, metabolic disorders, and chronic inflammation.^118^ However, in clinical practice, differences in dietary culture and patient compliance pose challenges to the application of this dietary strategy.^119^ Adaptation to local food cultures is therefore critical.

#### 4.1.3. Plant‑based diet

A plant-based diet centers primarily on foods of plant origin (such as whole grains, legumes, vegetables, fruits, nuts, and seeds), while limiting or excluding animal-derived products to varying degrees.^120^ Plant-based diets hold potential value for recovery following antibiotic treatment, not only due to increased dietary fiber intake but also a diverse array of phytochemicals and micronutrients that help restore the gut microbial ecosystem. Plant diversity supports a wider range of microbial niches and metabolic functions, thereby promoting the long-term stability and resilience of the gut microbiota community. The diet is rich in dietary fiber, resistant starch, polyphenols, and micronutrients, and is generally low in saturated fat. This overall nutrient profile is generally favorable to beneficial gut microbiota.^121^ In particular, plant-derived substrates can promote the growth of *Bifidobacterium* and other fiber-fermenting taxa, while limiting microorganisms that are better adapted to high-fat, low-fiber dietary conditions.^122^

By reducing the intake of animal-derived protein and increasing the diversity of plant-derived substrates, a plant-based diet may guide the microbiome's metabolism from proteolytic fermentation to glycolytic metabolism, thereby reducing the accumulation of pro-inflammatory metabolites such as ammonia, branched-chain fatty acids, hydrogen sulfide, and phenols.^123^ Their phytochemical content could provide additional support for mucosal recovery. Foods rich in polyphenols, flavonoids, and glucosinolates may help reduce oxidative stress in the gut, suppress pro-inflammatory responses,^124^ and support mucosal recovery through effects on tryptophan metabolism and AhR signaling.^125^ A recent study compared a plant-based enteral nutrition formula with a conventional enteral nutrition formula, showing that the plant-based diet was superior to the conventional formula in promoting the recovery of antibiotic-induced dysbiosis in mice and humans. In the clinical study of critically ill children, children receiving the plant-based formula showed increased gut commensal bacteria, reduced pathogens, and improved systemic immune cell balance compared with children receiving the conventional formula.^122^ These findings suggest that plant-based dietary patterns may offer a promising ecological framework for post-antibiotic microbiota recovery, although further direct clinical studies are still needed.

It is worth noting that the diversity and integrity of plant-derived substrates are key determinants of a plant-based diet. Highly processed versions that are heavy in refined carbohydrates and low in intact fiber and polyphenols may have little impact on microbial recovery.^126^ This makes a focus on whole-food plant-based dietary patterns, rather than simply on whether a diet contains animal products, an important part of dietary guidance.

**Figure 4.**
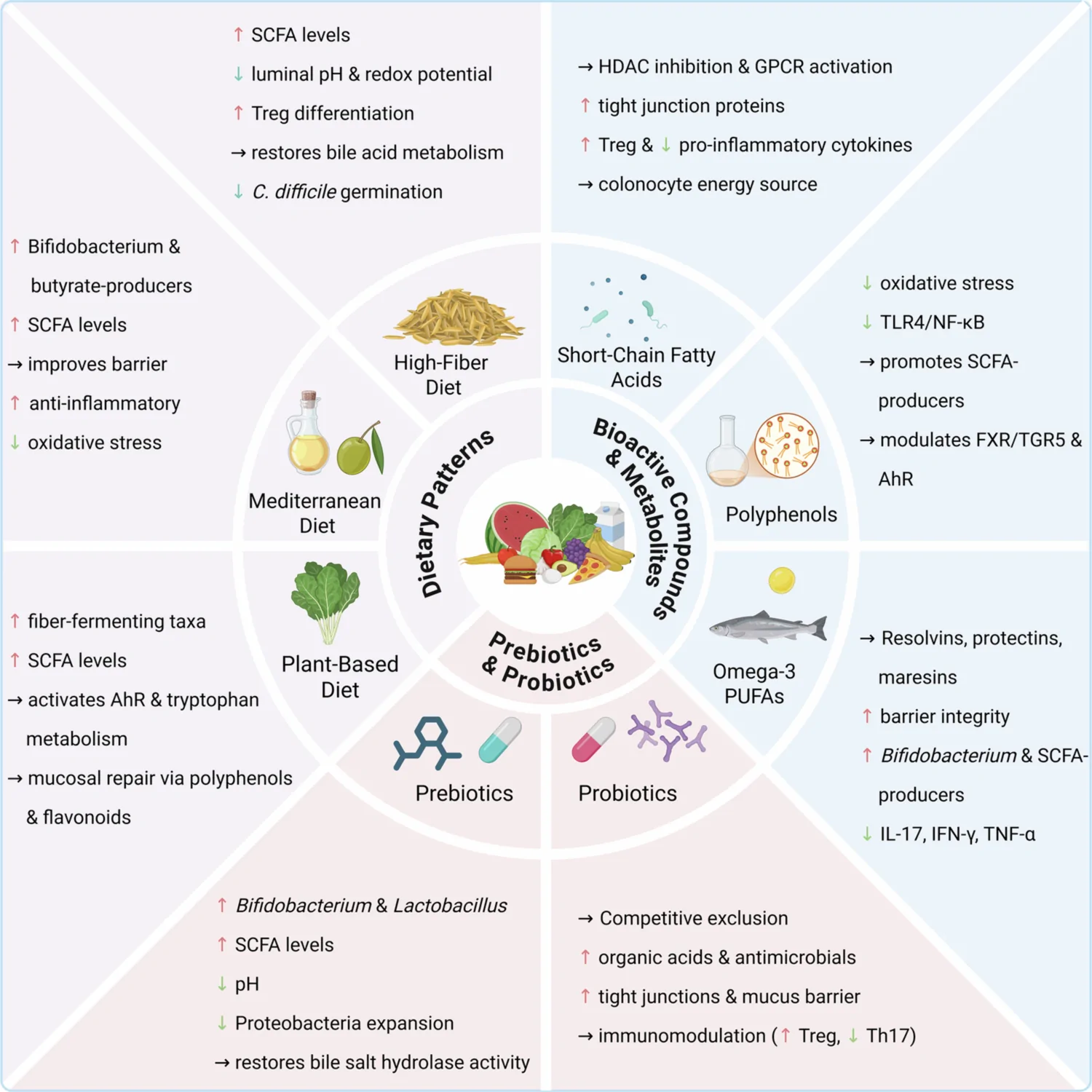
Nutritional strategies for post-antibiotic recovery of gut ecosystem.

In summary, while high-fiber, Mediterranean, and plant-based diets all share the common feature of fiber intake, they differ in their primary mechanisms of action. The high-fiber diet is primarily a substrate-oriented intervention defined by the type and fermentability of microbiota-accessible carbohydrates. Its most direct effect is to replenish substrates for fermentative microbes, thereby supporting SCFA production and the restoration of other fermentation-related functions. In contrast, the Mediterranean diet is a multi-component, holistic dietary pattern. Beyond fermentable carbohydrates, this dietary pattern is characterized by a high intake of polyphenol-rich foods, olive oil, and other sources of unsaturated fats. Consequently, it may influence recovery from the disruption caused by antibiotic treatment through synergistic effects on bile acid metabolism, oxidative stress, and the host inflammatory response. Plant-based diets are characterized primarily by the diversity of plant-derived foods. Their potential value lies not only in increasing fiber intake but also in expanding the variety of phytochemicals and micronutrients available to restore the gut ecosystem. Greater plant diversity can support a wider range of microbial niches and metabolic functions, thereby contributing to long-term community stability and resilience. Therefore, these dietary approaches should be viewed as complementary rather than mutually exclusive. Further research is needed to determine the relative efficacy and optimal timing for implementing these dietary strategies (Figure 4).

### 4.2. Prebiotics and probiotics

#### 4.2.1. Prebiotics

Prebiotics are substrates that the gut microbiota can selectively use, with benefits for host health. Typical prebiotics include inulin, fructooligosaccharides (FOS), galactooligosaccharides (GOS), resistant starch, and a range of fermentable dietary fibers. After antibiotic exposure, prebiotics may support recovery by serving as fermentable substrates for residual beneficial microbes. This may help restore populations of *Bifidobacterium*, *Lactobacillus*, and other butyrate-producing bacteria, and can also increase SCFA production.^127^ After the use of antibiotics, the levels of SCFA in the gut will significantly decrease. This decrease is considered a key intermediate step leading to a range of problems, including epithelial cell energy depletion, intestinal barrier disruption, increased permeability, and mucosal immune imbalance.^42^ Therefore, prebiotics have a significant advantage in helping to restore intestinal metabolism and barrier function after antibiotic withdrawal. Their role is not limited to restoring fermentation metabolism but also includes regulating the intestinal microenvironment. A recent study suggests that dietary fiber supplementation can mitigate antibiotic-induced microbial dysbiosis by lowering the redox potential of the gut. This limits the proliferation of opportunistic pathogens adapted to oxidative environments while promoting the restoration of strict anaerobic symbiotics.^106^ Meanwhile, the fermentation process of prebiotics can lower the pH of the intestinal lumen and reshape the competitive landscape of substrates. These changes can limit the abnormal proliferation of Proteobacteria after antibiotic withdrawal and promote the reconstruction of the anaerobic symbiotic network between Firmicutes and Bacteroidetes.^106^ Since microbiota with bile salt hydrolase activity and secondary bile acid production capacity tend to decrease after antibiotic treatment, prebiotics may also indirectly improve bile acid homeostasis and reduce the risk of CDI.^128^

People respond to prebiotics in very different ways. Several factors are likely to influence the response, such as the starting microbiota profile, the nature and intensity of previous antibiotic exposure, host nutritional status, and the degree of intestinal inflammation.^129^ When microbiota depletion is pronounced, especially with the loss of key functional taxa, prebiotics by themselves may have limited capacity to restore microbial function.^130^ In practice, this means prebiotics should probably be part of a wider dietary plan. For more severe dysbiosis after antibiotics, prebiotics may need to be paired with other microbiome-directed approaches.

#### 4.2.2. Probiotics

Probiotics are defined as live microorganisms that, when administered in adequate amounts, provide a health benefit to the host. Available evidence suggests that they may contribute to gut homeostasis through several mechanisms. They compete with pathogens for nutrients and space, limiting their ability to colonize the gut.^131^ Many strains also produce lactic acid, other organic acids, and antimicrobial compounds that can suppress pathogen growth.^132^ Some probiotics appear to support restoration of the mucus barrier and improve tight junction integrity.^133^ Probiotics may also influence both innate and adaptive immune responses. There is also evidence that *Clostridium scindens* and *Bifidobacterium longum* help maintain the microbiota's ability to metabolize tryptophan and convert bile acids.^48^ The strains most frequently studied are include *Lactobacillus* and *Bifidobacterium* species, the yeast *Saccharomyces boulardii*, and various multi-strain combinations.^134^

Probiotics are among the most extensively studied dietary interventions for the prevention of antibiotic-associated diarrhea (AAD) and CDI. Pooled data from systematic reviews and meta‑analyzes show that specific probiotic formulations can moderately reduce AAD rates and lower the risk of CDI in some populations.^135^ ^,^ ^136^ Several mechanisms have been proposed, including enhancing colonization resistance,^137^ attenuating *C. difficile* virulence,^138^ and reducing the ecological space of pathogens following antibiotic-associated dysbiosis.^48^ *S. boulardii* is often given attention because most antibiotics do not directly inhibit it.^139^ In contrast, the *Lactobacillus* and *Bifidobacterium* strains are more effective in breaking down substrates and can produce SCFA precursors.^140^

The effects of probiotics differ among strains, and there are significant differences between different strains. Their clinical efficacy depends not only on the strain itself, but also on factors such as dosage and the host's original gut microbiota.^141^ Many probiotic strains fail to establish stable reproduction in the gut after antibiotic treatment, resulting in minimal benefit for some patients. In some cases, exogenous probiotics may even delay the natural reconstruction of the gut microbiota. This indicates that probiotic supplements should not be simply regarded as a superior alternative to restoring endogenous gut microbiota.^142^ These findings suggest that the selection of strains should be based on evidence regarding the specific strain's mechanism of action and the clinical characteristics of patients (Table 3).

**Table 3.** Studies on probiotic intervention in antibiotic-associated diseases.

Study	Disease type	Strain	Dose	Experimental type	Mechanism of action	Key effects
Buffie et al. [48]	Antibiotic-induced CDI	*Clostridium scindens* ATCC 35704	1 × 10^6^ CFU/day, gavage, 2 days	Mouse models	7α-dehydroxylation restores secondary bile acids (DCA, LCA) that directly inhibit *C. difficile*.	Reduced *C. difficile* burden and toxin; improved survival; restored secondary bile acids.
Zhang et al. [143]	Antibiotic-induced intestinal inflammation	*Lactobacillus reuteri* SKLAN202402ZF	8.1 × 10^6^ CFU/day, gavage, 2 weeks	Piglet & mouse models	Inhibits TLR4/MyD88/NLRP3; reduces IL-1β, IL-18; upregulates tight junction proteins.	Ameliorated intestinal damage and inflammation; restored barrier integrity; attenuated weight loss.
Zhao et al. [144]	Antibiotic-induced entero-vaginal dysbiosis	*Limosilactobacillus reuteri* NCU-15 (oral); *Lactobacillus crispatus* NCU-23 (intravaginal)	NCU-15: 1 × 10^8^ CFU/day, 14 d; NCU-23: 5 × 10^4^ CFU/day, 14 d	Mouse models	NCU-15: inhibits gastric NF-κB, restores intestinal barrier. NCU-23: inhibits vaginal TLR4/MyD88/NF-κB, reduces apoptosis.	Oral NCU-15 ameliorated gastritis, restored gut barrier and Lactobacillus. Intravaginal NCU-23 reduced vaginal pathogens, increased Lactobacillus, reduced inflammation. Dual therapy synergistically restored both niches.
Goldenberg et al. [145]	Antibiotic-induced CDI	Multiple genera (Saccharomyces, *Lactobacillus, Bifidobacterium, Streptococcus*)	Varied across 31 RCTs	Meta-analysis of 31 RCTs (*n* = 9955)	Presumed restoration of colonization resistance and barrier function.	Reduced CDI risk (RR 0.40; NNT = 40); greater benefit in high-risk settings (RR 0.30; NNT = 12). Reduced antibiotic-associated diarrhea (RR 0.58).
Foley et al. [137]	Antibiotic-induced CDI	*Lactobacillus gasseri* Lg-36; *Lactobacillus acidophilus* NCFM	1 × 10^8^ CFU, single gavage post-antibiotics	Mouse models	*L. gasseri*: putative bacteriocin inhibits *C. difficile*; enriches *Muribaculaceae* that compete for mucosal sugars. *L. acidophilus* impaired colonization resistance.	*L. gasseri* reduced *C. difficile* burden, accelerated colonization resistance; Muribaculaceae bloom cleared *C. difficile* at week 3. L. acidophilus increased *C. difficile* burden, delayed recovery.
Merenstein et al. [146]	Antibiotic-induced fecal SCFA reduction	*Bifidobacterium animalis* subsp. lactis BB-12	1 × 10^10^ CFU/day, 14 d (yogurt)	RCT (*n* = 62 healthy adults)	Attenuates antibiotic-induced SCFA reduction; protects against gut microbiota taxonomic disruption.	Fecal acetate returned to baseline by day 30 (−1.6% vs −25.1% control); smaller decrease in Shannon diversity; fewer loose stools (26% vs 44% control).
Schepper et al. [147]	Antibiotic-induced bone loss	*Lactobacillus reuteri* ATCC PTA 6475	3.3 × 10^8^ CFU/mL drinking water + 1 × 10^9^ CFU gavage 3 × /week, 4 weeks	Mouse models	Reduces Firmicutes, Bacteroidetes ratio; prevents intestinal barrier disruption; reduces colonic TNF-α/IL-10 ratio.	Prevented trabecular bone loss; reduced osteoclast markers, increased osteoblast markers; prevented intestinal permeability increase.

### 4.3. Metabolite‑targeted interventions and bioactive compounds

#### 4.3.1. Short-chain fatty acids

Short-chain fatty acids (SCFAs), particularly acetate, propionate, and butyrate, are central to gut metabolic homeostasis, barrier integrity, and immune balance. Antibiotic exposure can significantly reduce the production of SCFAs, and this reduction effect may persist even after the treatment is completed.^148^ Butyrate activates GPR41, GPR43, and GPR109A and inhibits histone deacetylases (HDACs). These effects enable butyrate to enhance the expression of tight junctions, stabilize the mucus barrier, and promote the differentiation of Treg.^149^ Directly adding butyrate or its precursors is a feasible approach, and it may provide a relatively rapid way to restore the availability of SCFA.^150^ However, low delivery efficiency, difficulty in precisely targeting specific areas of the intestine, and limitations in tolerability restrict clinical application. Therefore, researchers are exploring methods such as improved encapsulation technologies and sustained-release systems to overcome these limitations.^151^ Another strategy is to promote SCFA generation by increasing fermentable substrates, such as dietary fiber, resistant starch, and selected prebiotics. This approach more closely reflects normal physiological fermentation and may be better suited for long-term maintenance of SCFA production.

The SCFA metabolic axis plays a central part in antibiotic-associated dysbiosis. Nutritional strategies that target its restoration are therefore attracting growing interest. The evidence to date comes mainly from animal models, in vitro experiments, and small human trials. Still, several strategies have produced encouraging results in antibiotic-exposed models and populations. These include high-fiber diets, prebiotics, probiotics, and synbiotics. In animal models, dietary fiber appears to support SCFA-producing communities indirectly by preserving anaerobic conditions and protecting commensal microbiota.^106^ Some prebiotics are also showing promise. For example, 2′-fucosyllactose (2′-FL) temporarily improved gut microbiota resilience and lowered plasma interleukin-6 (IL-6) levels in adults exposed to vancomycin.^152^ Supplementation with *B. animalis* subsp. *lactis* BB-12 has also been shown to partially preserve fecal acetate levels and speed their recovery after amoxicillin-clavulanate treatment. By contrast, direct SCFA supplementation has been studied much less extensively.^146^ In animal models of colitis, extended-release butyrate has partially restored *Clostridia* populations and shown both anti‑inflammatory and barrier‑protective effects.^153^ In post-antibiotic recovery, SCFA restoration may be more appropriately viewed as a functional goal than as an intervention in isolation.

#### 4.3.2. Polyphenolic compounds

Polyphenols constitute a large and diverse family of plant-derived secondary metabolites, comprising over 8,000 identified compounds that are broadly classified into four major groups: flavonoids (such as flavonols, flavones, flavanols, anthocyanins, and isoflavones), phenolic acids (such as hydroxybenzoic and hydroxycinnamic acids), lignans, and stilbenes. They are abundant in berries, grapes, tea, cocoa, olives, and many traditional plant-based foods.^154^ Polyphenols are poorly absorbed in the small intestine, with only about 5%–10% taken up before reaching the colon. Most dietary polyphenols therefore reach the colon, where gut microbiota breaks these compounds down into smaller metabolites such as phenolic acids and urolithins. Some of these microbial products are absorbed more easily, and some carry greater biological activity than the parent compounds.^155^ Despite structural diversity, polyphenols share a common capacity to modulate the gut microbiota by promoting the growth of beneficial taxa such as *Bifidobacterium*, *Lactobacillus*, and *Akkermansia*, while suppressing opportunistic pathogens including *Escherichia-Shigella* and *Clostridium* species.^156^ However, different classes of polyphenols exhibit different mechanistic characteristics: flavonoids can effectively restore the Firmicutes/Bacteroidetes ratio and enhance the production of SCFAs; anthocyanins are metabolized by gut microbiota into simpler phenolic metabolites, which have antioxidant and anti-inflammatory effects; phenolic acids can regulate the expression of pro-inflammatory cytokines.^157–159^ This diversity of chemical structures and interactions with microorganisms endow polyphenols with broad-spectrum effects in the gut after antibiotic treatment, and can be used to support the restoration of gut microbiota and mucosal repair.

In the setting of antibiotic-associated dysbiosis, polyphenols help restore the mucosal environment and the metabolic networks that microbial recovery depends on. By mitigating antibiotic-induced mucosal injury, oxidative stress, and local inflammation through antioxidant, anti-inflammatory, and barrier-protective actions, polyphenols may improve the local intestinal milieu and reduce the risk of progression to chronic inflammation and metabolic impairment. These changes may provide a more permissive intestinal niche for the recolonization of obligate anaerobic commensals. In experimental models of antibiotic-induced dysbiosis, apple peel polyphenols have been shown to upregulate tight junction proteins and suppress Toll-like receptor 4 (TLR4) and nuclear factor-κB (NF-κB) signaling, thereby reducing intestinal inflammation and epithelial injury.^160^ Tea polyphenol supplementation has also been associated with the recovery of taxa such as *Lactobacillus*, *Akkermansia*, *Blautia*, and *Roseburia*, together with increased SCFA levels.^161^ In addition to protecting the intestinal barrier, polyphenols may affect host signaling through microbiota-dependent changes in microbial composition and metabolism. These effects may involve farnesoid X receptor (FXR)/TGR5- and AhR-related pathways, as well as microbial processes such as SCFA production, bile acid transformation, and tryptophan metabolism, all of which are relevant to long-term metabolic and inflammatory outcomes.^125^ Clinical evidence for the direct reparative effects of polyphenols after antibiotic exposure remains limited. Yet polyphenol-rich dietary patterns, especially the Mediterranean diet and other traditional plant-based diets, remain a sensible, practical choice for supporting gut microbiota recovery.

#### 4.3.3. Omega‑3 polyunsaturated fatty acids

Omega-3 polyunsaturated fatty acids (*ω*-3 PUFAs) are a class of dietary functional lipids. They include alpha-linolenic acid (ALA) and its long-chain derivatives eicosapentaenoic acid (EPA) and docosahexaenoic acid (DHA). Major dietary sources of these fatty acids include deep-sea fish, fish oil, algal oil, flaxseed, perilla seed, and walnuts. Unlike polyphenols, which are mainly converted by colonic microbes, *ω*-3 fatty acids can be directly absorbed and utilized by the host.^162^ Their main nutritional value lies in several aspects, including regulating inflammatory responses, maintaining cell membrane fluidity, and generating specific pro-remission lipid mediators. Studies have shown that *ω*-3 fatty acids can competitively inhibit the synthesis of pro-inflammatory mediators derived from arachidonic acid, while promoting the generation of specific pro-resolving molecules, including resolvins, protectins, and maresin. They can also enhance the integrity of the intestinal epithelial barrier, reduce oxidative stress levels, and regulate innate and adaptive immune responses.^163^ Increasing evidence suggests that *ω*-3 fatty acids can indirectly influence the composition of the gut microbiota and the production of its metabolites by altering the inflammatory environment in the intestinal lumen and the metabolic state of the mucosa. As a result, specific anti-inflammatory microbiota and SCFA-producing bacteria are promoted.^164^ Therefore, in the interaction framework of dietary lipids-host inflammation-gut microbiome, *ω*-3 fatty acids occupy a key nutritional node position, possessing the dual characteristics of host regulation and microbiome support.

The value of ω‑3 fatty acids in antibiotic-associated dysbiosis lies not in directly replacing the functions of lost microbes, but in reshaping the host's own inflammatory environment and barrier microenvironment. In this way, they can reduce the risk of dysbiosis escalating into persistent inflammation and metabolic disorders.^165^ Randomized controlled trials have shown that ω‑3 fatty acid supplementation can alter the composition of the gut microbiota in healthy adults and significantly increase the abundance of several SCFA-producing bacterial groups.^166^ In mice with colitis lacking interleukin-10 (IL-10), DHA can reduce the degree of epithelial damage and downregulate the levels of pro-inflammatory cytokines such as IL-17, IFN-*γ*, and tumor necrosis factor-*α* (TNF-*α*).^167^ Under physiological conditions, EPA‑ and DHA‑rich fish oil interventions have been shown to increase the abundance of *Bifidobacterium*, *Lactobacillus*, and *Akkermansia*, while also strengthening the colonic mucus barrier and raising butyrate levels.^168^ These findings suggest a possible role for *ω*-3 fatty acids in recovery after antibiotic treatment. Their effects on mucosal inflammation and epithelial repair may help re-establish local immune balance. In turn, this may facilitate the return of anaerobic commensal bacteria and the recovery of microbial metabolic activity.^165^ Although evidence from large-scale clinical trials remains limited, current findings suggest that *ω*-3 fatty acids could be useful as a post-antibiotic dietary intervention, particularly because of their anti-inflammatory, barrier-protective, and microbiome-modulating properties.

#### 4.3.4. Other bioactive compounds

In addition to polyphenols and PUFAs, other dietary bioactive compounds, particularly polysaccharides and alkaloids, have also been reported to alleviate antibiotic-induced dysbiosis in animal models.

Among these additional bioactive compounds, polysaccharides have been studied most extensively. Despite their diverse structures, ranging from marine sulfated polysaccharides (such as fucoidan,^169^ fungal or medicinally derived polysaccharides (such as CSP-1^170^ and DIPY,^171^ and plant-derived polysaccharides (such as KRG-*P* ^172^ and PSPP,^173^ these compounds share some common mechanisms in antibiotic-induced dysbiosis. In different experimental systems, they are generally associated with increased microbial diversity and abundance, enrichment of SCFA-producing bacteria (such as *Blautia*, *Bifidobacterium*, and the Lachnospiraceae), and suppression of pathogenic bacteria-associated taxa (particularly Enterobacteria and Proteobacteria). These microbiota changes are typically accompanied by decreased expression of pro-inflammatory cytokines, increased expression of IL-10, and improved intestinal barrier integrity through repair of mucosal structures and tight junction proteins. Meanwhile, significant differences exist among the polysaccharides. Fucoidan may have a more direct protective effect against antibiotic-induced bacterial growth inhibition, while polysaccharides from plants and fungi are more often used as substrates for fermentation, supplementing SCFAs to achieve immunomodulatory effects. Their bioactivity appears to be closely associated with structural features,^174^ including the degree of sulfation, monosaccharide composition, and molecular weight.

Alkaloids are a diverse class of nitrogen-containing secondary metabolites. Their primary mechanism of action in regulating gut ecology is through immunomodulation and metabolic pathway regulation, rather than through substrate fermentation or structural protection as seen with polysaccharides. The evidence comes from the total alkaloids of *Corydalis saxicola* Bunting (TACS), which restored antibiotic-disturbed genera in rats, including *Blautia* and *Intestinibacter*, and modulated multiple metabolic pathways (including branched-chain amino acid, bile acid, aromatic amino acid, and amino sugar metabolism).^175^ Molecular docking experiments further demonstrated that TACS acts on CYP27A1, a key enzyme in bile acid synthesis. However, structural differences among alkaloids lead to distinct effects; for example, berberine has been shown to protect intestinal integrity and modulate gut microbiota and bile acid metabolism,^176^ whereas certain analogs such as sanguinarine and chelerythrine can enrich Proteobacteria and significantly reduce butyrate production.^177^ Therefore, the efficacy of alkaloids is closely tied to their structural features, with different subclasses exhibiting distinct effects on microbial composition and host metabolism.

## 5. Conclusion

A convergence of epidemiological, mechanistic, and clinical evidence demonstrates a clear association between antibiotic-induced gut microbiota dysbiosis and increased risk of a variety of diseases. This evidence spans a broad timeframe, from the long-term effects of perinatal and early childhood exposure on obesity, allergic diseases, and metabolic dysfunction, to increased risk of CDI, IBD, and other gastrointestinal disorders in adulthood. All of this underscores the central role of the gut microbiota in host health. Nutritional strategies, as a non-pharmacological and non-invasive approach, are theoretically very attractive. These strategies aim to mitigate so-called “antibiotic scarring” and reduce subsequent disease risk by selectively modulating the structure and function of the microbiota using dietary components. These strategies aim to restore several key pathways, including SCFA production, bile acid metabolism, and the function of the tryptophan-AhR axis. Among these strategies, probiotics have accumulated the most clinical evidence. They have shown relatively consistent benefits in preventing AAD and have also reduced the risk of CDI in certain high-risk populations. However, it should be noted that under certain conditions, particularly when the microbiota is severely depleted, probiotic administration may impede endogenous reconstitution, highlighting the need for strain-specific and context-dependent application. Comprehensive dietary patterns such as high-fiber diets and the Mediterranean diet primarily benefit by integrating and modulating microbial diversity, inflammatory responses, and the metabolic environment. These advantages make them well-suited for long-term management in the real world. Currently, the clinical value of these nutritional strategies is most evident in two areas: preventative support and rehabilitation adjuncts.

Translating nutritional strategies into routine clinical practice is not easy. A fundamental reason is the variability in individual responses. Baseline differences in gut microbiota composition and metabolic capacity can lead to varying outcomes, ranging from significant benefits to no effect at all. Multi-omics tools may eventually help identify who might benefit, but they are currently difficult to integrate into routine care. Patient adherence to nutritional strategies in the long term is equally important for actual effectiveness. The current evidence base also has limitations. Most clinical studies focus on relatively short-term results, such as antibiotic-associated diarrhea, CDI, or restoration of microbial diversity. We still lack direct evidence that these interventions reduce the long-term risk of diseases such as obesity or allergies. Large-scale, multicenter randomized controlled trials are needed to answer these questions. In the future, more advanced technologies and clearer guidelines may make nutritional interventions easier to implement precisely. For example, data models that can predict individual gut microbiota responses can help design personalized nutritional strategies. Combining narrow-spectrum antibiotics with targeted dietary intervention strategies may also help build treatment pathways that are more respectful of the gut ecosystem. Strict control over antibiotic use is also necessary to reduce antibiotic-related health risks. The next phase of nutritional intervention research needs to consider its effectiveness in daily life, not just in clinical trials, and assess its cost-effectiveness and intergenerational impact. Only in this way can the value of these interventions be fairly evaluated.

## Data Availability

No data was used for the research described in the article.

## Author statement

During the preparation of this work, the authors used DeepSeek in order to polish the language and improve the readability of the manuscript. After using this tool, the authors reviewed and edited the content as needed and take full responsibility for the content of the published article. The schematic figures were created with BioRender.com.
